# Hyaluronic acid-based nanofibers: Electrospun synthesis and their medical applications; recent developments and future perspective

**DOI:** 10.3389/fchem.2022.1092123

**Published:** 2022-12-23

**Authors:** Humaira, Sayyad Ali Raza Bukhari, Hafiz Abdullah Shakir, Muhammad Khan, Shagufta Saeed, Irfan Ahmad, Khursheed Muzammil, Marcelo Franco, Muhammad Irfan, Kun Li

**Affiliations:** ^1^ Department of Biotechnology, University of Sargodha, Sargodha, Pakistan; ^2^ Institute of Zoology, University of the Punjab New Campus, Lahore, Pakistan; ^3^ Institute of Biochemistry and Biotechnology, University of Veterinary and Animal Sciences Lahore, Lahore, Pakistan; ^4^ Department of Clinical Laboratory Sciences, College of Applied Medical Sciences, King Khalid University, Abha, Saudi Arabia; ^5^ Department of Public Health, College of Applied Medical Sciences, Khamis Mushait Campus, King Khalid University, Abha, Saudi Arabia; ^6^ Department of Exact Science and Technology, State University of Santa Cruz, Ilhéus, Brazil; ^7^ School of Medicine, Dalian University, Dalian, China

**Keywords:** hyaluronic acid, nanofibers, electrospinning, tissue engineering, wound healing

## Abstract

Hyaluronan is a biodegradable, biopolymer that represents a major part of the extracellular matrix and has the potential to be fabricated in a fibrous form conjugated with other polymers *via* electrospinning. Unique physicochemical features such as viscoelasticity, conductivity, and biological activity mainly affected by molecular weight attracted the attention of biomedical researchers to utilize hyaluronan for designing novel HA-based nano-devices. Particularly HA-based nanofibers get focused on a diverse range of applications in medical like tissue implants for regeneration of damaged tissue or organ repair, wound dressings, and drug delivery carriers to treat various disorders. Currently, electrospinning represents an effective available method for designing highly porous, 3D, HA-based nanofibers with features similar to that of the extra-cellular matrix making them a promising candidate for designing advanced regenerative medicines. This review highlights the structural and physicochemical features of HA, recently cited protocols in literature for HA production *via* microbial fermentation with particular focus on electrospun fabrication of HA-based nanofibers and parameters affecting its synthesis, current progress in medical applications of these electrospun HA-based nanofibers, their limitations and future perspective about the potential of these HA-based nanofibers in medical field.

## 1 Introduction

A unique, naturally occurring biopolymer “hyaluronan” (hyaluronic acid, HA) has received great attention in the biomaterial, bioengineering, and medical industry because of its exceptional physicochemical and biological features (Collins and Birkinshaw 2013). Biocompatibility, high moisture absorption capability, viscoelasticity, and excellent hygroscopic nature enable HA to be used as a joint structure stabilizer, shock absorber, lubricant, maintain water balance in the skin, and also serve as a flow resistance regulator (Necas et al., 2008; Liu et al., 2011; Ström et al., 2015). Key functions of HA include regulation of tissue hydration, cell adhesion, proliferation, and directed differentiation, and other responses such as wound healing, reducing inflammation, angiogenesis, and damaged tissue regeneration that makes HA an ideal candidate for bioengineering and designing novel devices for diverse applications in the medical field (Kang et al., 2011; Gayathri et al., 2018; Sihan et al., 2019). Thus, the medical and pharmaceutical industry particularly get focused on HA and constantly searching for new opportunities to optimize the production of economical, high-quality biocompatible, biodegradable HA for designing novel HA-based nano-devices for applications such as skin substitutes (Debels et al., 2015), novel wound dressings (Wang et al., 2022), drug carriers (Hosseini et al., 2021) cataract surgery, cancer treatment (Snetkov et al., 2019), tissue regeneration (Niu et al., 2021a) and hydrophilic membranes for postoperative tissue adhesion (Chen et al., 2021).

Advancement in regenerative medicines particularly relied upon designing novel 3D biocompatible nanostructures such as nanofibers (NFs) with an average diameter of <1000 nm. Currently, available synthetic tissue engineering constructs do not possess appropriate mechanical and structural features to provide an effective micro-environment to promote proliferation and cell adhesion; thus, biopolymers particularly the part of extracellular matrix (ECM) like hyaluronan (HA), collagen, fibronectin, etc. got attention and research for designing easy, cost-effective way for fabricating nanomaterials based on them is still in progress (Li et al., 2017; Tenchurin et al., 2021). Although researchers have developed several HA-based nanodevices of medical significance such as nano-hydrogels (Bhattacharya et al., 2019), liposomes (Choi et al., 2017), sponges (Niyama and Kuroyanagi, 2014), composites (Karimi et al., 2019), and nanoparticles (Fouda et al., 2016). This review particularly focuses on the promising medical applications of electrospun HA-based NFs. The unique features of NF include the 3D structure with high chemical stability, large aspect ratio, desired mechanical strength, loading efficiency, and controlled release capability enabling their widespread use in the biomedical field (Garg et al., 2015; Snetkov et al., 2019). For example, for tissue engineering, NFs are incorporated along-with seeded cells while in wound dressing high porosity of nanofibrous mat reported to improve the localized drug release which in turn may promotes the natural healing process (Jannesari et al., 2011; Ahadian et al., 2017).

Several techniques are used for nanofiber fabrication such as template synthesis (Zahmatkeshan et al., 2018), centrifugal spinning (Rim et al., 2013), drawing (Cheng et al., 2008), air jet spinning (Ashammakhi et al., 2009), phase separation (Taghavi and Larson, 2014), self-assembly (Asmatulu and Khan, 2019), melt spinning (Rim et al., 2013), electrospinning (aligned, random, core-shell NFs) (Samimi et al., 2018). Among these methods, electrospinning is an advance, relatively simple, easy way to generate micro and nanofibers of biopolymer solution. An electrospinning biopolymer such as HA solution is subjected to an electrostatic force which in turn generates porous, finished NFs with excellent surface adhesion capacity (Habibi et al., 2019). Processing conditions and biomaterial being used are key factors affecting NF’s morphology (Hajinasrollah et al., 2019). This review highlights the structural and physicochemical features of HA, natural sources, and recently reported protocols for industrial production of HA *via* microbial fermentation and HA-based NFs fabrication through electrospinning. Moreover, this review also reported the parameters affecting the fabrication process and morphology of NFs, medical applications of these electrospun NFs with particular focus on the recent progress in designing biocompatible HA-NFs-based tissue-engineering constructs and wound dressings to aid wound healing, limitations associated with toxic effects and future perspective regarding their wide-spread applications in biomedical industry.

## 2 Structural and physicochemical properties of HA

### 2.1 Structural features

**FIGURE 1:**
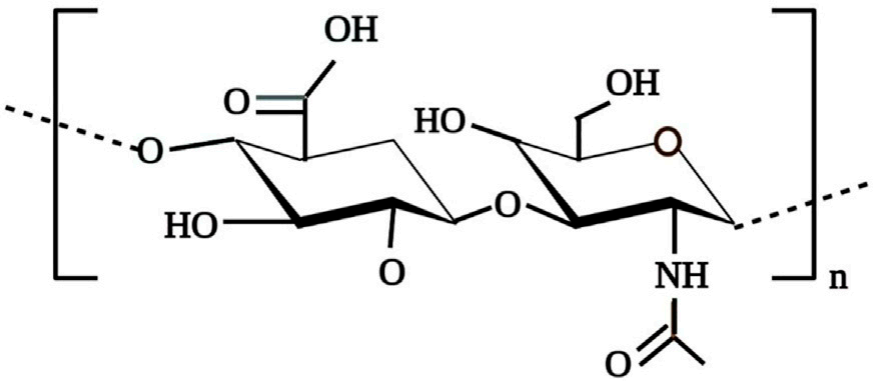
Chemical structure of HA.

HA is a non-sulphated, anionic and the simplest glycosaminoglycan (GAGs) comprised of *N*-acetylglucosamine and D-glucuronic acid repeating units up to 10,000 or more linked *via* β-1,3 and β-1,4-glycosidic bonds as shown in Figure 1 (Xu et al., 2012; Xing et al., 2020). HA, ability to retain large quantities of water is attributed to the abundance of the anionic hydroxyl group in the HA structure (Huang and Huang, 2018; Xing et al., 2020). This negatively charged gel-like polymeric structure not only permit HA to act as a lubricating agent for joints but also contributes to its shock-absorber ability around surrounding tissues (Xing et al., 2020). Presence of these polar and non-polar groups in the HA polymer permit it to chemically interact with other molecules such as chitosan, metachromatic dyes that results in the formation of polyelectrolyte complexes which in extend its applications in the biomedical field (Ma et al., 2012; Sridharan and Shankar, 2012).In solution, long-chain HA polymer exists in poly-dispersed form and possesses a 3D, viscous, random structure with high hydration volume that permits the free passage of micro-molecules through it but restricts the penetration of other macromolecules into the HA domain thus, contributes to the excluded volume effects of HA (Bastow et al., 2008; Xing et al., 2020).

### 2.2 Rheology

HA solution shows unique rheological behavior and retains its significance not only in medical, cosmetics, and bioengineering applications but also in biochemical and physiological processes and is primarily dependent on the HA structure and polyelectrolyte behavior of HA solution (Snetkov et al., 2020a). In general, the Hyaluronan solution displays viscoelastic, non-Newtonian behavior with shear-thinning (Fallacara et al., 2018; Kim et al., 2018). Various factors contribute to the shear-thinning behavior of HA solution such as increment in hydrophobic effects with enhanced shear rate and intramolecular hydrogen bond disruption. The hydrophobic effect is mainly generated by the implication of deformed HA molecular chains in flow direction which in turn reduce solution viscosity (Snetkov et al., 2020b). Falcon et al. (2006) have reported a reduction in untwining time of the 3D HA network by decreasing the molecular weight of hyaluronan. Generally, these rheological features of HA such as high viscoelasticity and high surface tension along with high hydrophilicity hinder the electrospinning of HA solution (Zhao et al., 2015). Furthermore, low vaporability and high conductivity of HA polymeric solution may cause circuit failure between needle and collector, thus limiting the efficacy of the electrospinning process (Snetkov et al., 2019).

### 2.3 Effect of molecular weight of HA on biological activity

Molecular mass and the HA synthesis and degradation conditions are the key factors affecting the biological activity of HA (Cyphert et al., 2015; Heldin et al., 2019). Generally, HMW (High molecular weight) and LMW (Low molecular weight) HA perform opposite biological functions (Girish and Kemparaju, 2007; Heldin et al., 2019). HMW hyaluronan (>10^6^ g/mol) is mainly present in synovial joints as it is highly viscoelastic and thus, acts as a lubricating agent and offers protection to articulate cartilage (Tamer 2013). Moreover, it displays anti-angiogenic activity and also plays a useful role in wound healing, tissue repair, immunosuppression, and inflammation by regulating the recruitment of inflammatory cytokines, inflammatory cells, fibrinogen binding, and stem-cell migration (Girish and Kemparaju, 2007; Jiang et al., 2011). While LMW hyaluronan (2 × 10^4^–2 × 10^6^ g/mol) can induce tumor progression by enhancing ECM remodeling (Cyphert et al., 2015; Wu et al., 2015). LMW HA also has the potential to stimulate the secretion of growth factors, chemokines, and proinflammatory cytokines (Heldin et al., 2019). Moreover, different studies have also evaluated the effect of HA’s molecular weight on the wound healing process. It was identified that HMW HA prevents apoptosis, enhances cell quiescence, contributes to the inflammatory phase of healing, maintains tissue integrity, and stimulates *in-vitro* proliferation of fibroblast (Selders et al., 2017; Sasaki et al., 2011)

## 3 Natural sources of HA

HA is widely distributed in humans and other animals such as roosters comb. In the human body, HA is primarily present in ECM of connective tissues, the pericellular coating around the cells, skin layers (about 50%) including both dermis and epidermis, eye vitreous body (0.1 mg/ml), synovial joints that contain approximately 3–4 mg/ml (wet weight) of hyaluronan (Schiraldi et al., 2010) and in the umbilical cord (4 mg/ml) where along-with chondroitin sulphate it represents a major constituent of Wharton’s jelly (Robert et al., 2010). HA is also naturally synthesized by bacteria such as *Streptococcus zooepidemicus, Streptococcus equi., Streptococcus equisemilis, Streptococcus uberis, Streptococcus pyogenes* and *Pasteurella multocida* (Schiraldi et al., 2010; De-Oliveira et al., 2016). All of these natural producers are known pathogens that produce endotoxins. Thus, researchers are focusing on the development of endotoxin-free alternative expression systems for HA production such as *Baccili* or *Escherichia coli* (Sze et al., 2016). Moreover, HA has also been produced by *Cryptococcus neoformans* (De-Olivera et al., 2016), mollusks (Volpi and Maccari, 2003) and algae such as *Chlorovirus* infected *Chlorella sp*. (De-Olivera et al., 2016).

## 4 Industrial production of HA

HA has received extraordinary attention in the industrial sector primarily because of its unique physiochemical features such as biocompatibility, viscoelasticity, biodegradability, hygroscopicity, lubricity, non-immunogenicity, mucoadhesive features, and biological functions like anti-inflammatory, wound healing and immunosuppressive effects. Hence, there is growing interest in developing systems for optimized production of cost-effective high-quality, HMW HA (Fallacara et all., 2018). The first industrial protocol developed for HA production involved the extraction of HA from animal sources. Although improved extraction protocols have been developed with time, animal-derived HA still poses several technical issues such as harsh purification conditions, low concentration, high degradation rate due to endogenous hyaluronidases, high polymer intrinsic dispersity, and risk of contamination which in turn requires processing *via* costly purification techniques (Boeriu et al., 2013; De-Oliveira et al., 2016; Knopf-Marques et al., 2016). Thus, alternative methods were searched and currently, commercial production of HA mainly relied upon microbial fermentation. Initially, Streptococci strains A and C were employed for the industrial production of HA. Nowadays, various HA-based products such as Juvederm^®^ by Allergan and Restylane^®^ by Q-med AB are commercially produced *via Streptococcus equi.* HMW HA (3.5–3.9 × 106 Da) was produced at a rate of 6–7 g/L by optimizing the bacterial culture parameters at 37°C, pH 7, and in the presence of sucrose or lactose (Rangaswamy and Jain, 2008). Some recently cited protocols in the literature for HA production through microbial fermentation are enlisted in Table 1.

**TABLE 1:** Some recently cited protocols in literature for microbial production of HA *via* fermentation.

Bacteria	Culture medium	Culture conditions	Mode-of fermentation	HA yield	References
*Streptococcus zooepidemicus* 39920	(g/L), Glucose 50, MgSO_4_ 0.5_,_ yeast extract 20, KH_2_PO_4_ 2, K_2_HPO_4_ 2, (NH_4_)_2_SO_4_ 0.5, ZnCl_2_ 0.046_,_ CaCl_2_ 2, CuSO_4_.5H_2_O 0.019, sucrose modified-FeNPs 30	37°C, pH 7, 200rpm	Batch	0.226 g/L	Wang et al. (2021)
*Streptococcus zooepidemicus* CCT 7546	1 ml of 50% glycerol culture stock solution, 50 ml of BHI broth, 0.9% NaCl (w/v)	37°C, 22 h, 150rpm	Batch	69.8 mg/L	Kimilly et al. (2022)
*Streptococcus zooepidemicus* (mutated through UV & N-methyl-N′-nitro-N-nitroguanidine)	(g/L) Yeast extract 20, Hydrolyzed casein 20, primary glucose 30, NaCl 1.5, MgSO_4_ 0.6 900 ml deionized water	37°C, pH 7, 5% O_2_ content	Fed-batch	8.4 g/L	Saharkhiz and Babaeipour, (2021)
*Streptococcus equi. Subsp. zooepidemicus* 3523	TH broth, 5 g/L brain, heart infusion	37°C, 22 h, 150rpm	Fed-batch	4.73 g/L	Mohan et al. (2022)
*Streptococcus zooepidemicus* ATCC 39920	(g/L): Glucose 25, Yeast extract, non-descript “salts” component 11	2VVM Airflow, 37°C, pH 7	Batch, fed-batch	2.5 g/L in batch, 5 g/L in fed-batch after 24 h	Ferreira et al. (2021)
*Streptococcus equi. Subsp. zooepidemicus*ATCC 35246	Molasses (6–20%), sheep wool proteins (SWP)	37°C, pH 8, 200rpm	Batch	3.54 g/L	Pinar and Nuri, (2021)
*Streptoccus Thermophillus TISTR 458*	(g/L): Glucose 30, NaCl 2, yest extract 30, K_2_HPO_4_ 2.5, MgSO_4_. 7H_2_O 1.5 g/L, sugarcane molasses	pH 6.8, 37 ± 2°C, 72 h	Batch	32.80 ± 4.27 (after 24 h) from glucose, 213.44 ± 76.79 mg/L (after 12 h) from molasses	Saraphanchottiwitthaya and Siripalakit, (2022)
*Streptococcus equi. Subsp. zooepidemicusATCC 35246*	(g/L): MgSO_4_. 7H_2_O 1.5, K_2_HPO_4_ 2.5, NaCl 2, yeast extract 10, monosaccharides in Bored coffee beans hydrolysates 30 (either acid or enzymatic hydrolysis)	37°C, 3000 rpm, 5% dissolved O_2_, 1 VVM airflow	Batch	2.7 g/L	Antonio et al. (2021)
*Streptococcus equi subsp. zooepidemicus*ATCC 39920	(g/L): sucrose 10–50, glutamate 0–0.6, yeast extract 10–50, oxalic acid, 0–0.6, glutamine 0–0.6, K_2_HPO_4_ 2.5, NaCl 2, MgSO_4_. 7H_2_O 1.5	150 rpm, 37°C, 48 h, 10% (VV^−1^)	Batch	0.860 g/L	Caldas et al. (2020)

## 5 Electrospinning process

**FIGURE 2:**
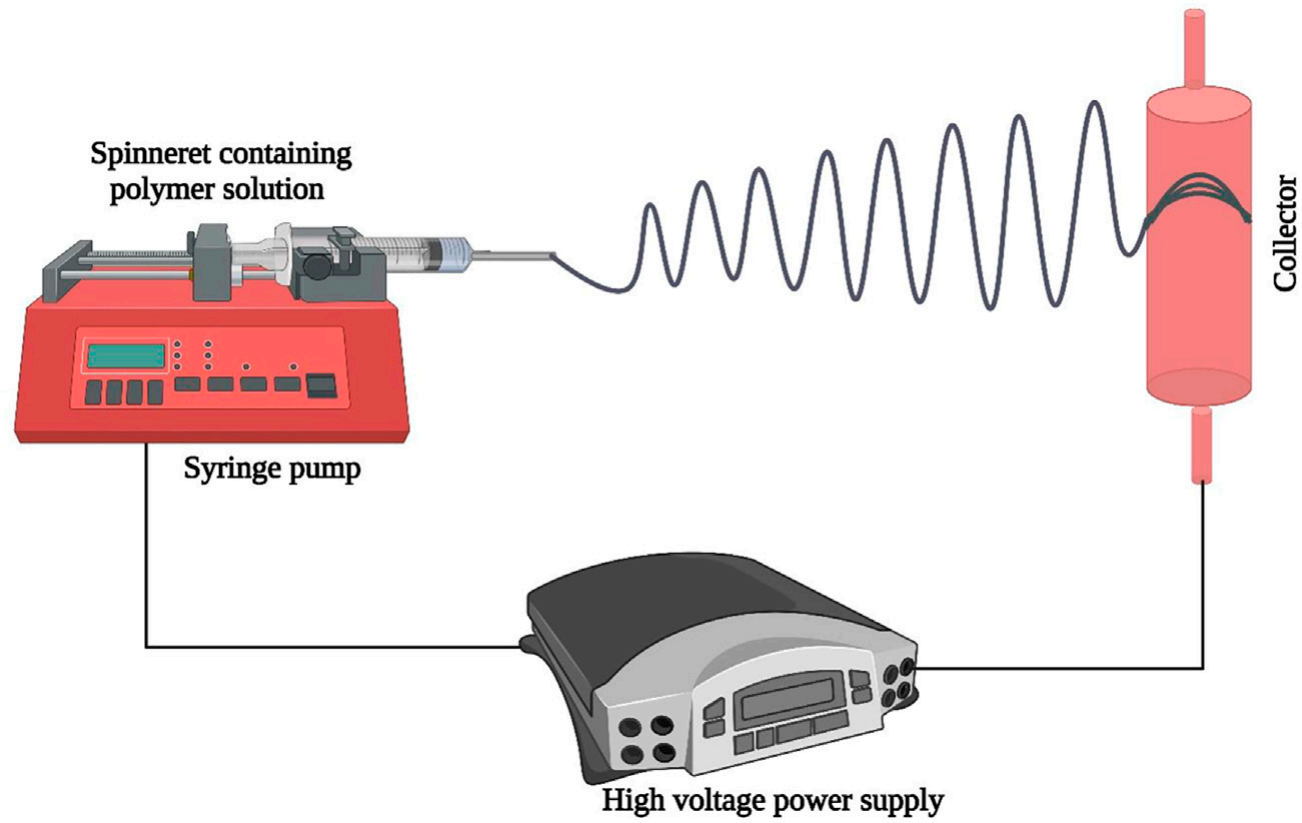
An overview of electrospinning system.

Electrospinning is a versatile, cross-sectional technique employed for the synthesis of polymers derived from micro and NFs that have found a diverse range of promising applications in the medical and pharmaceutical fields (Balusamy et al., 2017; Selders et al., 2017; Uyar and Kny, 2017). The electrospinning process involves the exposure of polymer solution to electrostatic forces and the production of a finished fibrous network thus, it is also termed hydrodynamic jetting (Jiang et al., 2015). A schematic representation of the electrospinning system is shown in Figure 2. The key component of electrospinning involves a delivery system for subjecting polymer solutions to electrostatic force. The syringe pump carries out this function and effectively delivers polymer solution to the metal needle spinneret. The second key constituent of electrospinning is a high voltage power supply (1–50 kV) applied between the spinneret and collector (Fahimirad and Ajalloueian, 2019; Memic et al., 2019; Snetkov et al., 2019).

A collector is the third central component that is a grounded, metal collecting electrode and is available in various forms such as disk, drum, plate, and mandrel. When high voltage is applied, a positively charged elongated polymer solution beam is ejected as a whip jet from the spinneret nozzle. During passage towards the collector electrode solvent evaporates and solidified network of polymer nanofiber is collected (Abrigo et al., 2014; Kalaf et al., 2017; Snetkov et al., 2019). Electrospinning of HA solution poses some challenges such as insolubility of HA in organic solvents which are usually preferred for electrospinning, probability of short-circuiting between collector electrode and spinneret needle because of high electrical conductivity of HA solution and low rate of evaporation. Advance electrospinning systems can detect any unusual circumstances and stop the process automatically (Selyanin et al., 2015).

### 5.1 HA-based NF fabrication *via* electrospinning

Several studies have reported the fabrication of HA-based NFs *via* electrospinning. For example, Snetkov et al. (2020a) have fabricated stable curcumin/usnic acid-loaded HA NFs *via* electrospinning of 1.9% (by wt.) HA solution containing curcumin/usnic acid and water-DMSO (di-methyl sulfoxide) solution at the volume ratio of 50:50. Scanning electron microscopy (SEM) images revealed the mean NFs diameter of 298 nm. Electric voltage was reported to be the most influencing factor during electrospinning and stable fiber was obtained at the applied voltage of 20–22 KV. However, some irregular morphologies i.e., beads, twisting, and drops that appeared at this voltage were reported to be greatly reduced at an applied voltage of 26–28 KV (Snetkov et al., 2020a). Authors reported that the possible occurrence of residual DMSO in electrospun NF can increase the antiseptic, analgesic, and anti-inflammatory activity of HA-NFs (Snetkov et al., 2020b). Similarly, Salim et al. (2021) have synthesized highly porous, 3D HA/PVA (Polyvinyl alcohol) mats through electrospinning. Electrospinning conditions were optimized, and stable fiber was obtained at a voltage supply of 30 KV and distance of 15 cm between the collector electrode and syringe-nozzle. Furthermore., the feeding rate of HA/PVA solution was reported to be a key factor that influences the topography of NF mat and uniform fiber was generated by optimizing the feeding rate at 0.3 ml/h. After collection, residual solvent from the NF mat was removed by heating in a dry oven for 2 h at 60°C. The biocompatibility of synthesized PVA/HA NFs was further improved by incorporating chitosan and hydroxyapatite (HAP) into the fibrous mat. Authors reported that the incorporation of chitosan greatly increased the swelling index, antimicrobial activity, protein adsorption, and hemocompatibility of PVA/HA NFs. While the incorporation of HAP significantly enhanced the thermal/mechanical stability of PVA/HA NF mats (Salim et al., 2021).

Moreover, PVA/Chi/HA NFs were also effectively fabricated *via* electrospinning (Hosseini et al., 2021). The polymeric solution was subjected to electrospinning at an applied voltage of 19 kV, 15 cm distance between collector and syringe needle, and a pump feeding rate of 0.8 mm/h. NF stability was further improved by exposing fibrous material to glutaraldehyde vapors (serve as crosslinking agent) for 3 h. SEM analysis showed that the mean diameter of NF before and after crosslinking was 261 ± 17 and 288 ± 20 nm. Authors reported that PVA/Chi/HA NFs exhibited significant potential for controlled release of hGA (human growth agent) with an initial burst release rate of 11% followed by gradient release of hGA up to 64% within 48 h (Hosseini et al., 2021). Niu et al. (2021a) have reported the fabrication of a porous, hierarchical, tubular HA/collagen nanofiber scaffold by electrospinning. 2% HA and 10% collagen solution were injected at the rate of 0.36 ml/L at a voltage supply of 11 KV. Electrospinning was carried out at 18–25°C, 40–45% relative humidity for 20 h and 60 h for HA and collagen solution respectively. Tensile strength and stability of HA/collagen NF scaffolds were further improved by exposing composite NF scaffolds to 2.5% glutaraldehyde vapors for 6 h that serve as crosslinking agents followed by sterilization under a UV lamp for 2 h. These double-layered HA/collagen NFs have tightly layered outer walls and loose inner layers with a mean diameter of 905 ± 113 nm. *In-vitro* evaluations revealed that the highly porous outer surface of tubular NF scaffold can promote the photomorphogenesis of vascular smooth muscles (SMs) *via* diffusion and infiltration, while the inner wall can promote the adhesion of vascular endothelial cells (ECs) (Niu et al., 2021b). Abou-Okeil et al. (2021) have reported the fabrication of novel electrospun HA/oxidized-K-carrageenan (OKC) nanofibers. Polymer solution (1% HA and OKC and 10% PVA solution) was injected into the electrospinning system at the rate of 0.5 ml/h, at 25°C ± 2°C, and voltage supply of 17.5 KV. The finished electrospun NF was collected at a distance of 10 cm from the syringe needle. Authors reported that obtained HA/OKC NF mats exhibited good antibacterial activity against both Gram -ve (*E. coli*) and Gram + ve (*S. aureus*) bacteria (Abou-Okeil et al., 2021).

## 6 Medical applications of HA-derived nanofibers

Currently, bioengineering and biomedical research get focused on designing biomolecules derived nanofibrous structures as the majority of human tissues (cartilage, bone, skin, etc.) exist in nanofibrous form with organized hierarchical structure (Khadka and Haynie, 2012; Selyanin et al., 2015). As HA is a central component of ECM, thus; HA-based electrospun NF has porosity, surface area, and diameter like ECM and can be a promising approach for advancement in tissue engineering, wound healing, and localized drug release. Here, recent progress in designing novel HA-based tissue implants and wound dressings is enlisted.

### 6.1 HA nanofibers as wound dressings:

**FIGURE 3:**
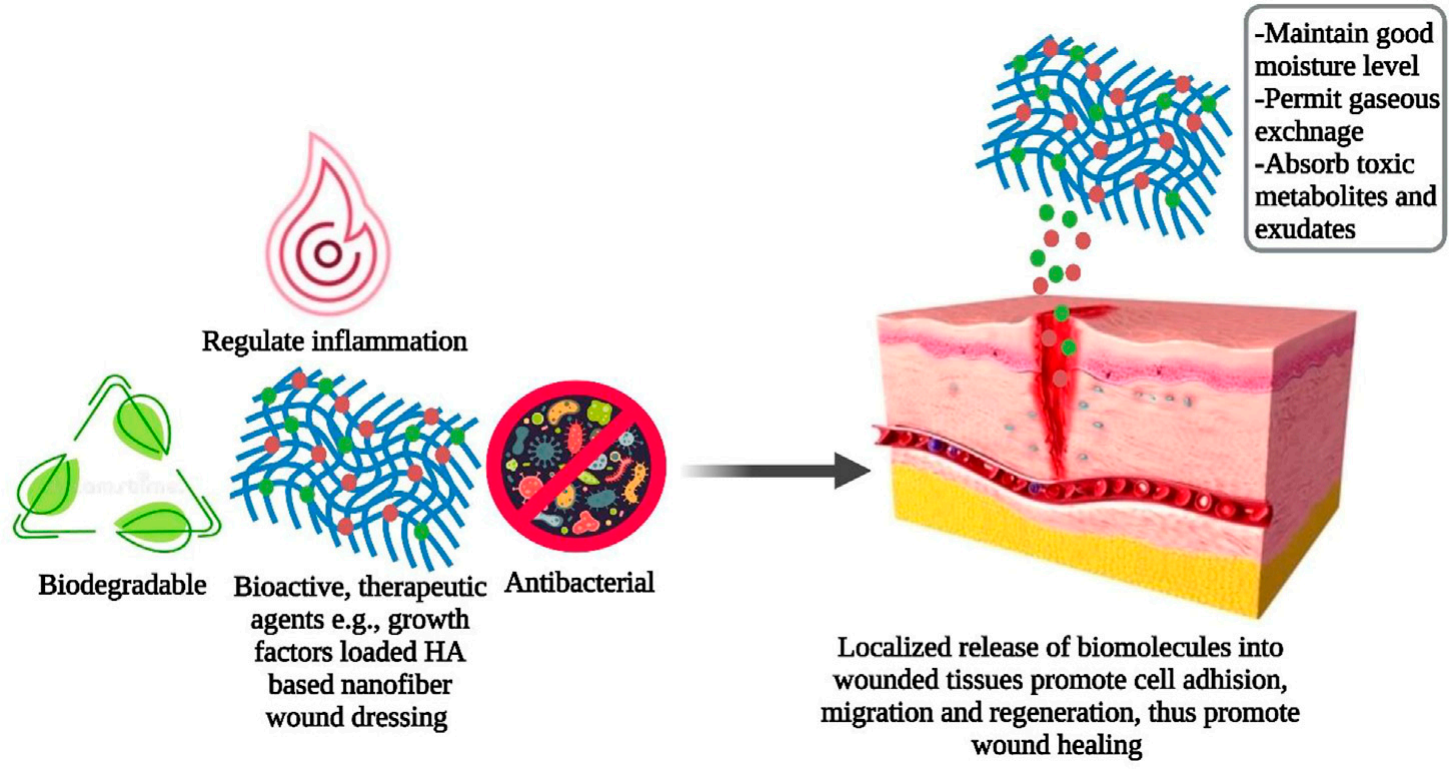
Mechanism adopted by HA-based wound dressings for wound healing.

Biomedical research is constantly searching for new opportunities to promote cost-effective wound healing therapy (Veith et al., 2019). During the inflammatory phase of the healing process, the limited number and brief life span of neutrophils make wounds highly susceptible to bacterial attack which in turn limits the efficacy of the healing process (Bjarnsholt 2013). Ideal wound dressings should maintain appropriate moisture levels, permit gaseous exchange, and absorb toxic metabolites and surplus exudates to promote cell growth and differentiation that ultimately aid in the natural healing process (Barbosa et al., 2016; Debone et al., 2019). The absence of bioactivity and biodegradability, inconvenient cleaning, hemostasis during replacement, and Potential source of allergenicity and immunogenicity make traditional wound dressings clinically less desirable (Tang et al., 2019; Ying et al., 2019). Hence, engineered biomaterials such as NF membranes have received attention as their flexible, large, porous, 3D networks identical to ECM promote biological functionalization and chemical modifications making them an ideal candidate for modern wound dressings (Chen et al., 2018). Moreover, biodegradable, biopolymers (e.g., fibroin, HA, chitosan) derived NFs as wound dressings permit incorporation and localized release of biomolecules that enhance cell adhesion, migration, and regeneration as shown in Figure 3; thus, greatly expanding their applications in wound care management (Miguel et al., 2019; Jain et al., 2020; Stojanov and Berlec, 2020).

Various studies have reported the excellent wound healing potential of HA-derived electrospun NFs. For example, Wang et al. (2022) have fabricated berberine (BBR) loaded electrospun cellulose acetate-hyaluronic acid (CA/HA) NFs. These CA/HA/BBR NFs exhibited a tensile strength of 2.99 ± 0.05 MPa and elongation break of 47.8% with an average diameter of 502 ± 50 nm determined *via* SEM analysis. Wound dressings fabricated from these CA/HA/BBR NFs have displayed >95% antimicrobial efficiency against *E. coil* and *S. aureus*. Moreover, *in-vitro* incubation of L929 fibroblastic cells has shown a highly augmented proliferation rate and cell viability (>99) after 7 days. *In-vivo* evaluations of CA/HA/BBR NF-based bandages in rats revealed reduced wound size, enhanced collagen development, and healing potential presenting them as promising wound dressing candidates for cutaneous wound healing (Wang et al., 2022). Mahmood et al. (2022) evaluated the wound care potential of zinc and silver ion-loaded HA-alginate NFs. Significant bactericidal activity against *S. aureus* and *E. coli*, high tensile strength, gelation, and maximum absorption capacity of 26.6 g/g have shown the good wound exudate absorption ability of these Zn and Ag ions loaded HA-Alginate NFs (Mahmood et al., 2022). Yang et al. (2021) reported the fabrication of ԑ-polylysine (EPL) loaded NF mats (OHA-EPL). In contrast with control starch-EPL NFs, higher hydrophilicity of HA attributed to its anionic nature results in higher absorbance (26.3-times exudates), higher EPL content (from 19.2% to 27.9%), rapid fiber degradation, and improved tensile strength from 0.3 MPa to 0.6 MPa. Hence, along with the biological activity of HA, these OHA-EPL NF mats may possess better clinical wound treatment activity as they displayed broad-spectrum antibacterial activity, appropriate permeability, and good biocompatibility than starch EPL- NF mats (Yang et al., 2021).

Elibol et al. (2021) have evaluated the vocal cord wound healing potential of HA-collagen NFs in white rabbits. Results of seventh day H&E staining, Masson trichome staining, and Van Gieson staining analysis have shown that HA-collagen NFs can be employed to treat impaired viscoelasticity because of fibrosis after tissue injury and impaired voice quality in disorders that are characterized by thickening of the propria layer in the vocal cord (Elibol et al., 2021). Moreover, crosslinked HA NFs fabricated *via* periodate oxidation-Adipic acid dihydrazide (ADH) crosslinking approach exhibited high tensile strength up to 0.88 MPa, excellent biocompatibility, absorbance, and water resistance over 14 days making the OHA-ADH nanofibrous mats a promising wound dressing candidate (Xue et al., 2021). El-Assar et al. (2020) have fabricated AgNPs embedded NFs composed of HA and polygalactuoronic acid (PGA) *via* electrospinning. Nanoscale electrospinning of (Ag-PGA/HA)-PVA NF was confirmed *via* SEM which displayed an average diameter of 326 nm. In (Ag-PGA/HA)-PVA NF, AgNPs serve as anti-inflammatory and antioxidant agents that speed up the healing process by offering protection against augmented ROS generation. While HA constituents contribute to strain activities and high hydrophilicity. *In-vivo* administration of this (Ag-PGA/HA)-PVA NF has shown maximum collagen deposition and wound epithelialization after 14 days (El-Aassar et al., 2020).

### 6.2 HA nanofibrous scaffold mediated tissue engineering

**FIGURE 4:**
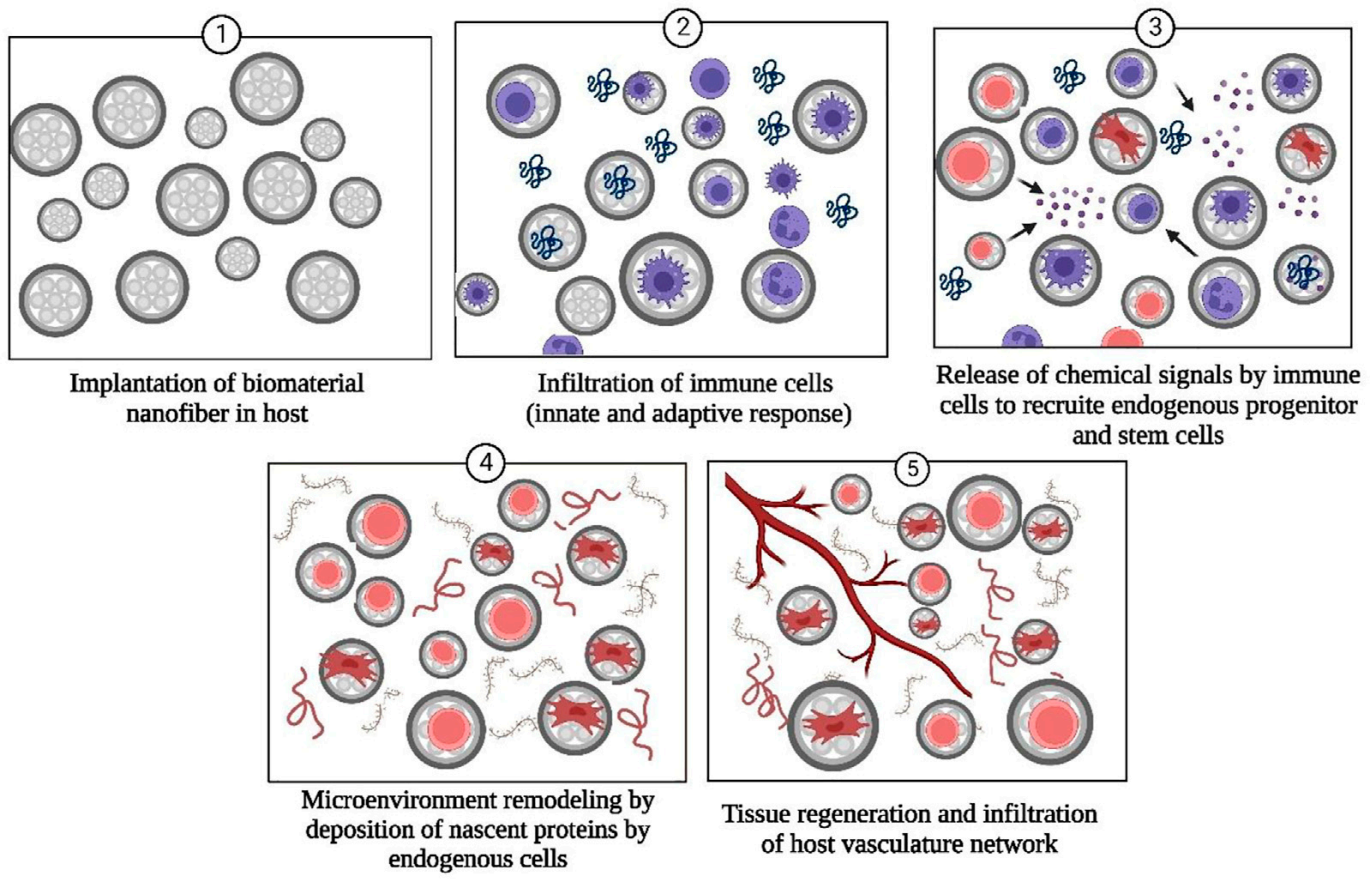
Mode of action of HA-based nanofiber scaffolds for tissue regeneration.

Tissue engineering represents an advanced method of treating the complications that arise during surgical treatments and a promising technique for organ regeneration as thousands of patients worldwide suffer from organ failures annually (Dzobo et al., 2018; Rasouli et al., 2019; Lanza et al., 2020). Tissue engineering involves growing desired cells and tissues *in-vitro* and then implanting them into the host organism (Dzobo et al., 2018). Currently, nanofibrous tissue-engineered scaffolds have received attention as the most appropriate way to support *in-vitro* grown cells/tissues. Along with the biocompatible and biodegradable nature of biomolecules-derived NF scaffolds, other factors that should also be considered while designing tissue engineering scaffolds include porosity, size, and tensile strength of biological material (Sridhar et al., 2011; Khanna et al., 2021; Sharifianjazi et al., 2021). Nanofibrous scaffolds provide an innovative class of materials as their fibrous, porous structure, high surface energy, and area in contrast with bulk material and similarity with the natural ECM and biological tissues permit increased adhesion, differentiation of cells, and unhindered transport of waste and nutrients (Freed et al., 2006). Unique features of HA such as immune neutrality (Andorko and Jewell 2017; Jin et al., 2020), ability to modify the mechanical strength of ECM (Ibrahim et al., 2010), high hydrophilicity (Gebe et al., 2017), biodegradability (Fuenteslopez and Ye, 2020), and capability to bind with cell surface receptors (e.g., ICAM, CD44) (Galarza et al., 2019; Amorim et al., 2021) that promotes cell motility make HA and its derivatives highly promising candidates for tissue regeneration and surgical implants. The mode of action of HA and biopolymer-based NF scaffolds for promoting tissue regeneration is schematically described in Figure 4.

Various studies have reported the excellent tissue regeneration capacity of HA-derived NFs. For example, Niu et al. (2021c) have evaluated the effectiveness of HA functionalized collagen NFs for urethral regeneration by modifying the pro-healing phenotype expression of macrophages. HA-collagen nanofibrous mats with HA coating were fabricated *via* coaxial electrospinning. HA coating provides better mechanical softness and higher anisotropic wettability to NFs. ELISA and immunofluorescence assays have demonstrated that elongated macrophages growing over HA-collagen nanofibers could potentially decrease the release of inflammatory cytokines and upregulate the expression of the M2 phenotype marker. Moreover, *in-vivo* administration in male puppies revealed the enrichment of recruited anti-inflammatory M2 macrophages over the nanofiber surface, thus generating signals that promote urethral regeneration through the proliferation of endogenous urethral progenitor cells and angiogenesis (Niu et al., 2021a). Salim et al. (2021) have synthesized chitosan and hydroxyapatite (HAP) incorporated polyvinyl alcohol-hyaluronan (PVA/HA) NF mats and evaluate their potential for bone tissue regeneration. Authors reported that chitosan incorporation significantly increases the antimicrobial activity and swelling index of PVA/HA nanofibrous mats. While the addition of HAP improves thermal and mechanical stability. Moreover, all the tested NF mats with variable composition have shown high cell viability independent of concentration and incubation time of nanofibers. *In-vitro* analysis revealed a high rate of WI38 cell adherence and proliferation over HAP-loaded PVA/HA NFs making them a promising biomaterial for bone tissue regeneration (Table 2) (Salim et al., 2021).

**TABLE 2:** *HA-derived nanofibers and their tissue engineering applications*.

Nanofiber’s material	Potential application	Results	References
HA-SF nanofiber scaffolds	Urethral regeneration	*In-vivo* administration show enhanced adhesion, proliferation, and growth of primary urothelial cells and increased expression of uroplakin-3. Thus, promoting luminal epithelialization and rapid reconstruction of the urothelial barrier in the wounded area	Niu et al. (2021b)
Aligned HA/PRP-PCL CSNFMs	Tendon tissue engineering	*In vitro* evaluations demonstrated enhanced cell proliferation, upregulated gene expression and marker protein synthesis, reduced tendon maturation time, and maintenance of tenogenic phenotype in contrast with static culture	(Chen et al. (2021a))
HA-PLA/AgNPs CSNFMs	Prevention of post-operative tendon adhesion	The *in-vitro* evaluation revealed that CSNFM possesses low cytotoxicity, significant antibacterial activity, prevents fibroblast penetration, and shows the highest efficacy in reducing fibroblast adhesion. While *in-vivo* analysis revealed anti-inflammatory potential and prevention from peritendinous adhesion	Chen et al. (2021b)
oHAs- modified collagen nanofibers	Vascular tissue engineering	oHAs-modified collagen nanofibers increase endothelial cell proliferation with no detectible coagulation and hemolysis which makes them a potential candidate for vascular tissue engineering	Kang et al. (2019)
Core-shell PLLA/HA nanofibers	Pelvic ligament tissue engineering	*In-vitro* evaluation of Core-shell PLLA/HA nanofibers on mBMSCs revealed no cytotoxic effects and enhanced cellular activity that is further confirmed by RT-qPCR analysis of Col1a1, Col1a3, and Tnc (pelvic ligament related gene markers)	Zhang et al. (2021)
Collagen/HA nanofibers	Vascular tissue engineering	Displayed potential for complete endothelialization of PAECs and structural remodeling of SMCs, with no detectable coagulation and hemolysis suggesting their potential as an engineered vascular tissue implant	Niu et al. (2021c)
HA/Carbon nanotubes (CNT) nanofibers	Neural engineering	Electrical stimulation *via* HA/CNT nanofibers effectively enhanced sustained neuron growth as confirmed *via* neuron number and neurite length after 72 h by applying 20 Hz biphasic AC waveform just for 1 hour	Elisabeth et al. (2019)
Col/oHAs-based nanofibers	Bone tissue engineering	*Invitro* culturing of PIEC and infiltration of MC3T3-E1 in hybrid nanofiber network significantly enhance cell adhesion, proliferation, and upregulated expression of OCN and ALP directing towards osteogenic differentiation	Li et al. (2019)
HepMAHA nanofibers	Sequestering GFs release in spinal cord injury	HepMAHA nanofibers loading into L929 fibroblasts in growth media significantly increase proliferation (*α* < 0.05) after 24 h. Moreover, the longest dissociated chick dorsal root ganglia neurite was reported in SEM.	Mays et al. (2020)
PCL/HA-based nanofiber scaffolds containing L-Ascorbic acid	Skin tissue engineering	Results demonstrated that nanofiber scaffolds increased the cell growth, proliferation, and adhesion of L929 fibroblast cells. Thus, PCL/HA nanofiber scaffolds containing 40 mg of AA could be applied for skin tissue engineering	Janmohammadi et al. (2021)

HA-SF, hyaluronic acid coated silk fibroin; HA/PRP-PCL, hyaluronic acid/platelet-rich plasma-polycaprolactone; CSNFMs, core-sheath nanofiber membranes; HA-PLA/AgNPs, hyaluronic acid-polylactic acid/silver nanoparticles; oHAs, hyaluronic acid oligosaccharides; PLLA/HA, poly (l-lactic acid)-hyaluronic acid; mBMSCs, mouse bone marrow-derived mesenchymal stem cells; Col/oHAs, collagen modified with hyaluronic acid oligosaccharides; PIEC- artery endothelial cells; PAECs, mouse primary aortic endothelial cells; SMC, smooth muscle cells; MC3T3-E1, mouse parietal bone cell; OCN, osteocalcin; ALP-alkaline phosphatase; SFM-serum-free media; HepMAHA, heparin methacrylate hyaluronic acid; PCL, polycaprolactone; L-AA- L, ascorbic acid.

Moreover, Martin et al. (2021), have fabricated nanofibrous HA scaffolds, which efficiently release Transforming Growth Factor-β3 (TGF-β3) and Stromal-Cell Derived Factor-1α (SDF-1α); thus, promoting cartilage tissue repair. *In-vitro* analysis has shown that dual factor release (TGF-β3 and SDF-1α) significantly enhances cell migration and improves matrix deposition by mesenchymal progenitor cells. While *in-vivo* evaluation in a large animal model to repair a large cartilage defect revealed that local release of SDF-1α impedes neo-cartilage tissue regeneration and thus, possesses lower cartilage healing potential in contrast with TGF-β3 that significantly increases cartilage tissue regeneration (Martin et al., 2021).

### 6.3 Electrospun HA nanofibers as drug delivery carrier

NFs have received great attention in the medical field as a carrier for localized drug release or other active substances. Usually, drugs or other active compounds are either blended with NFs or supplied in bound form by using one or more nanoparticles. Various studies have reported electrospun HA-based NFs as efficient localized drug release carriers. For example, Hosseini et al. (2021) have evaluated the efficacy of biocompatible electrospun PVA/Chi/HA NFs for sustained release of human growth hormone (hGH). Authors have reported that initial burst release of hGH (11%) occurs within the first 2 h followed by sustained release of hGH (64%) after 48 h (Hosseini et al., 2021). Initial burst release can be attributed to the hydrophilic nature of NFs and the presence of hGH molecules on NF’s surface. While later 64% hGH release from PVA/Chi/HA NF might be due to polymeric eruption (Hosseini et al., 2021). In another study, Seon-Lutz et al. (2018) have fabricated insoluble HA/PVA/HPβCD (hydroxypropyl-β-cyclodextrin) NFs *via* electrospinning in pure water. The drug release potential of these insoluble HA-based NFs was evaluated by using non-steroidal anti-inflammatory naproxen (NAP) as a model. NAP was impregnated into nanofibrous scaffolds either under super-critical CO_2_ or in an aqueous solution. The functional NFs revealed maximum drug release after 24 h while sustainable drug release potential of more than 48 h without damaging fibrous structure (Seon-Lutz et al., 2018).

## 7 Conclusion and future perspective

Currently, biomedical research is mainly focused on designing biocompatible, biodegradable, 3D, biopolymer-based nanostructures to overcome the challenges associated with synthetic nano-devices and to provide a microenvironment similar to that of natural ECM. These nano constructs show potential to treat medical dilemmas. Thus, biopolymers such as HA have attracted attention as it represents a key component of ECM and also possesses the ability to design fibrous structures at nanoscales for diverse medical applications. Various protocols have been developed for the economical, commercial production of HA. Although various studies have reported the fabrication of HA-based NFs still several technical issues such as circuit breaks and solution parameters such as high viscosity and conductivity of HA are required to be managed to improve the stability, tensile strength, and structural features like porosity which in regulate the biological activity of these NFs. Progress in designing optimized electrospun protocols and implantation strategies can diversify the range of medical applications of these biopolymer-based NFs.
